# A randomized placebo-controlled pilot study of *N*-acetylcysteine in youth with autism spectrum disorder

**DOI:** 10.1186/s13229-016-0088-6

**Published:** 2016-04-21

**Authors:** Logan K. Wink, Ryan Adams, Zemin Wang, James E. Klaunig, Martin H. Plawecki, David J. Posey, Christopher J. McDougle, Craig A. Erickson

**Affiliations:** Cincinnati Children’s Hospital Medical Center, University of Cincinnati College of Medicine, 3333 Burnet Avenue MLC 4002, Cincinnati, OH 45229 USA; Investigative Toxicology and Pathology, School of Public Health, Indiana University, Bloomington, IN USA; Department of Psychiatry, Christian Sarkine Autism Treatment Center, Riley Hospital for Children at Indiana University Health, Indiana University School of Medicine, Indianapolis, IN USA; Indianapolis, IN USA; Lurie Center for Autism, Departments of Psychiatry and Pediatrics, Massachusetts General Hospital and MassGeneral Hospital for Children, Harvard Medical School, Boston, MA USA

**Keywords:** Autism, Autism spectrum disorder, Social impairment, *N*-acetylcysteine, Oxidative stress

## Abstract

**Background:**

Social impairment is a defining feature of autism spectrum disorder (ASD) with no demonstrated effective pharmacologic treatments. The goal of this study was to evaluate efficacy, safety, and tolerability of oral *N*-acetylcysteine (NAC), an antioxidant whose function overlaps with proposed mechanisms of ASD pathophysiology, targeting core social impairment in youth with ASD.

**Methods:**

This study was a 12-week randomized, double-blind, placebo-controlled trial of oral NAC in youth with ASD. Study participants were medically healthy youth age 4 to 12 years with ASD, weighing ≥15 kg, and judged to be moderately ill based on the Clinical Global Impressions Severity scale. The participants were randomized via computer to active drug or placebo in a 1:1 ratio, with the target dose of NAC being 60 mg/kg/day in three divided doses. The primary outcome measure of efficacy was the Clinical Global Impressions Improvement (CGI-I) scale anchored to core social impairment. To investigate the impact of NAC on oxidative stress markers in peripheral blood, venous blood samples were collected at screen and week 12.

**Results:**

Thirty-one patients were enrolled (NAC = 16, placebo = 15). Three participants were lost to follow-up, and three left the trial due to adverse effects. The average daily dose of NAC at week 12 was 56.2 mg/kg (*SD* = 9.7) with dose ranging from 33.6 to 64.3 mg/kg. The frequency of adverse events was so low that comparisons between groups could not be conducted. At week 12, there was no statistically significant difference between the NAC and placebo groups on the CGI-I (*p* > 0.69) but the glutathione (GSH) level in blood was significantly higher in the NAC group (*p* < 0.05). The oxidative glutathione disulfide (GSSG) level increased in the NAC group, however only at a trend level of significance (*p* = 0.09). There was no significant difference between the NAC and placebo groups in the GSH/GSSG ratio, DNA strand break and oxidative damage, and blood homocysteine levels at week 12 (*p*s > 0.16).

**Conclusions:**

The results of this trial indicate that NAC treatment was well tolerated, had the expected effect of boosting GSH production, but had no significant impact on social impairment in youth with ASD.

**Trial registration:**

Clinicaltrails.gov NCT00453180

## Background

Social and communication impairments are the defining features and key predictors of long-term outcome in autism spectrum disorder (ASD) [1, 2]. To date, no medication has demonstrated significant impact on these core deficits in controlled trials. In 2014, the United States Center for Disease Control reported that one in 68 children in the USA is diagnosed with ASD, highlighting the critical need for targeted treatment development in this disorder [3]. Growing neurobiologic understanding of ASD has identified glutamatergic neurotransmission and metabolic pathways impacting oxidative stress levels as potential targets of drug development in this complex disorder [4]. Glutamatergic dysfunction appears to play a significant role in ASD pathology, as studies have identified abnormal peripheral glutamate levels, aberrant glutamate expression in the postmortem brain, and genetic abnormalities in glutamate signaling genes in individuals with ASD [5]. Excessive oxidative stress also has been identified as playing a potential role in ASD pathophysiology. Increased peripheral oxidative biomarkers, evidence of oxidative stress in the postmortem brain, and abnormalities in genes encoding for antioxidant enzymes have been reported in individuals with ASD [4, 6, 7].

*N*-acetylcysteine (NAC) is a unique antioxidant whose function overlaps with both the glutamatergic and oxidative stress hypotheses proposed to contribute to the pathophysiology of ASD. NAC is the *N*-acetyl derivative of l-cysteine used for decades in treatment of acetaminophen overdose, as a mucolytic in chronic obstructive pulmonary disease, and as a renal protectant in contrast-induced nephropathy [8]. NAC is rapidly absorbed via oral dosing, though total oral bioavailability of NAC is quite low (~9 %) [9]. NAC crosses the blood-brain barrier (BBB), though its efficiency may depend upon dose, administration, and formulation [10]. In the brain, NAC is oxidized from l-cysteine to cystine which is ultimately involved in regulation of extracellular glutamate levels [11]. NAC is cell membrane permeable and is reduced to cysteine intracellularly, which is a key component of the antioxidant glutathione (GSH) [10]. NAC’s involvement in both extracellular glutamate modulation and intracellular restoration of antioxidant GSH levels, coupled to its long history of human safety data, make NAC an intriguing substance in ASD-targeted treatment development.

Placebo-controlled studies of NAC in ASD have previously focused on treatment of ASD-associated irritability marked by physical aggression, self-injurious behavior, and severe tantrums. A pilot placebo-controlled study by Hardan et al. (2012) demonstrated a significant reduction in irritability symptoms as measured by the Aberrant Behavior Checklist Irritability subscale [12] (ABC-I) in 29 youth aged 3.2–10.7 years with ASD [13]. Additionally, two small double-blind, placebo-controlled studies of NAC in conjunction with risperidone for treatment of irritability in youth with ASD also showed a significant reduction in ABC-I subscale scores in the NAC-treated groups [14, 15]. Furthermore, case studies have demonstrated improvement of core ASD features including social communication and repetitive behaviors with NAC treatment; however, this effect has not been confirmed in controlled trials [16, 17].

To date, no study of NAC in ASD has evaluated the impact of NAC on markers of oxidative stress in peripheral blood. Individuals with ASD are believed to have decreased total GSH levels and elevated levels of oxidative glutathione disulfide (GSSG) [4]. These abnormalities lead to a reduction in the ratio of active GSH to inactive GSSH resulting in increased intracellular oxidative stress which may impact on individual’s capacity to maintain cellular methylation and increase vulnerability to oxidative damage [18]. This change has the potential to decrease an individual’s ability to resist infection, resolve inflammation, and respond to environmental exposures. Correction of these abnormalities with NAC treatment could have substantial impact on the health of individuals with ASD.

The goal of this randomized, double-blind, placebo-controlled pilot study of NAC in youth with ASD was to evaluate the efficacy of oral NAC targeting core social impairment of ASD and evaluate the safety and tolerability of NAC in this population. Additionally, this project incorporated measures of oxidative stress that may be impacted by NAC treatment, including measures of pre- and post-treatment peripheral whole blood levels of GSH and GSSG. We hypothesized that treatment with NAC in this population would result in improvement in core social impairment of ASD as measured by the Clinical Global Impression Improvement (CGI-I) scale [19], and that NAC treatment would result in increased GSH and GSSH levels.

## Methods

### Study design

This study was a 12-week randomized, double-blind, placebo-controlled pilot trial of oral NAC in youth with ASD. The study was conducted at Indiana University School of Medicine (IUSM) between December 2006 and November 2009. The study was approved by the IUSM institutional review board. Guardians of all participants provided written informed consent prior to study enrollment. Assent was obtained from enrolled youth when possible.

Study participants were youth ages 4 to 12 years with a diagnosis of autistic disorder, Asperger’s disorder, or pervasive developmental disorder not otherwise specified (PDD NOS). Subjects with known genetic syndromes associated with autism were excluded (for example, fragile X syndrome, tuberous sclerosis). Participants were diagnosed via clinical interview completed by study physician with expertise in ASD (DJP, CJM, CAE), based on the Diagnostic and Statistical Manual of Mental Disorders, Fourth Edition (DSM-IV) [20] diagnostic criteria, and corroborated by administration of the Autism Diagnostic Interview-Revised [21] (ADI-R). Participants diagnosed with PDD NOS demonstrated pervasive impairment in social interaction and/or communication skills as well as stereotyped behaviors and restricted interests, but did not meet full criteria for diagnosis of autistic disorder or Asperger’s disorder. All participants weighed ≥15 kg and were medically healthy based on physical exam and review of medical history completed by study physicians. Participants were judged by study physician as being at least “moderately ill” as measured by the Clinical Global Impression Severity [19] (CGI-S) scale rating at baseline. The CGI-S is a clinician-rated global assessment of symptom severity scale ranging from 1 to 7 (1 = normal, not at all ill; 2 = borderline ill; 3 = mildly ill; 4 = moderately ill; 5 = markedly ill; 6 = severely ill; 7 = among the most extremely ill patients). Concomitant medications were permitted if doses were stable for at least 60 days prior to study initiation and remained stable throughout the study. Participants taking known glutamatergic modulators such as dextromethorphan, d-cycloserine, amantadine, memantine, lamotrigine, or riluzole were excluded. Patients taking daily acetaminophen, daily nonsteroidal anti-inflammatory medications, daily antioxidant medications such as high-dose vitamin supplements, and medications with known drug-interactions (i.e., carbamazepine) within 30 days of baseline were also excluded. Subjects were required to be able to swallow capsules. Potential subjects with profound cognitive impairment (mental functioning below 18 months of age) as measured by the Leiter International Test of Intelligence-Revised [22] were excluded.

Following screening and baseline measures, participants were randomized 1:1 via computer—by the investigational pharmacy. All participants, guardians, and investigators remained blind to study assignment. NAC and matching placebo were prepared by CustomMed Apothecary. Participants randomized to active drug were administered capsules containing 300 or 600 mg of NAC, with a target dose of 60 mg/kg/day in three divided doses and a maximum dose of 4200 mg/day. Patients weighing 15 to 30 kg began treatment with a starting dose of 300 mg/day; those weighing above 30 kg started with 600 mg/day. Patients were required to tolerate a minimum dose of 300 mg/day to continue in the trial. Dose was titrated to the target dose over the first 3 weeks of study participation. Dose then remained stable for all subjects in the last 9 weeks of the study, although reductions due to adverse effects were permitted at any time. Subjects were evaluated in person at screen and baseline, by phone at week 2, and in person at weeks 4, 8, and 12. Assessment for adverse effects was completed during each interaction. Vital signs including height, weight, blood pressure, and heart rate were measured at every in-person study visit. Safety labs including complete blood cell count (CBC) and comprehensive metabolic panel (CMP) were collected at screen and week 12.

The primary outcome measure of efficacy was the CGI-I scale anchored to study physician (DJP, CJM, CAE) assessment of core social impairment considering the individuals’ overall level of cognitive, adaptive, and social functioning. The CGI-I is a clinician-rated global assessment of symptom change rated on a scale from 1 to 7 (1 = very much improved; 2 = much improved; 3 = minimally improved; 4 = no change; 5 = minimally worse; 6 = much worse; 7 = very much worse). In this study, a positive response to NAC treatment was defined as scoring a 1 “very much improved “or 2 “much improved” on the CGI-I. Study physicians completed annual CGI training to ensure internal consistency with this outcome measure. Secondary outcome measures included the CGI-S, ABC, Social Responsiveness Scale [23] (SRS) raw score, and Vineland Adaptive Behavior Scales 2nd Edition [24] (VABS-II) survey edition raw score. The SRS is a standardized, caregiver reported measure of the core symptoms of ASD [23]. The ABC is a parent questionnaire measuring five behavioral domains including irritability, social withdrawal, stereotypy, hyperactivity, and inappropriate speech [12]. The VABS-II is a semi-structured caregiver interview which provides a measure of an individual’s overall adaptive functioning [24]. All measures have been used extensively in ASD research [25–27]. The CGI-I was completed at weeks 4, 8, and 12. The ABC and SRS were completed at baseline and weeks 4, 8, and 12. The CGI-S and VABS-II were completed only at baseline and week 12.

Early morning, fasting, venous blood samples for measurement of oxidative stress biomarkers GSH, GSSH, total homocysteine, strand breakage, and oxidative DNA damage were collected in EDTA containing Vacutainers at screen and week 12.

### Measurement of reduced and oxidized glutathione

Concentrations of reduced and oxidized glutathione (GSH and GSSG) in whole blood samples were analyzed simultaneously using HPLC-electrochemical detection as described previously [28]. Briefly, 1 ml of blood sample was added to 0.5 ml precipitating solution containing 0.2 M perchloric acid and 100 μM EDTA, and vortexed briefly. After incubation at room temperature for 45 min, samples were centrifuged at 10,000*g* for 3 minutes. The resulting supernatants were filtered and injected into high-performance liquid chromatography (HPLC; Waters 2690) for analysis immediately or frozen in liquid nitrogen and stored at −80 °C before analysis. The separation of analytes was achieved on a reverse phase Symmetry C-18 column (5 μm, 150 × 4.6 mm; Waters). GSH and GSSG were detected using an ESA Coulochem II (ESA Inc. Chelmsford, MA) equipped with a guard cell. The potential settings for the detector were E1 450 mV, E2 900 mV, and guard cell 1000 mV. The amount of GSH and GSSG was calculated from the respective calibration curves, and the ratio of GSH/GSSG for each sample was also calculated.

### Measurement of blood homocysteine

Total homocysteine in whole blood sample was determined using an HPLC method as described previously with minor modifications [29, 30]. Briefly, 1 ml EDTA blood samples were lysed by vigorously shaking for at least 10 s after adding 10 μl Nonidet P40 (pure) and 10 μl citric acid monohydrate (2.5 M). The lysates were then centrifuged at 10,000*g* for 3 minutes at room temperature. Following reduction of the sample with tri-n-butylphosphine, precipitation of protein with perchloric acid and derivatization with ammonium 7-fluorobenzo-2-oxa-1,3-diazole-4-sulfonate, the samples were then analyzed using reversed-phase high-performance liquid chromatography (Waters Alliance 2695 separation module) followed by fluorescence detection (Waters 474 fluorescence detector) (Waters Corporation, Milford, MA).

### Direct and oxidative DNA damage: comet assay

Immediately after blood collection, whole blood (10 μl) was mixed with 0.5 ml RPMI 1640 containing 10 % FBS, 10 % DMSO, 1 mM deferoxamine, step-frozen and stored at −80 °C until analysis. The comet assay was performed as described previously [31, 32]. Briefly, 6 μl of blood was mixed with 70 μl 1 % low-melting-point agarose and applied onto comet slides (Trevigen Inc, Gaithersburg, MD). Cells were lysed, placed in alkali buffer, and then electrophoresed. Slides were stained with ethidium bromide, and 100 randomly selected nuclei/sample were evaluated (Komet 5.5; Kinetic Imaging Ltd., Liverpool, UK). For the assessment of oxidative DNA damage, a modified alkaline comet assay was performed that included enzymatic digestion with formamidopyrimidine-DNA glycosylase (fpg) prior to electrophoresis. DNA damage was expressed as Comet (Olive) tail moment [(tail mean − head mean) × tail%DNA/100].

### Statistical analysis

Our sample size (goal 32 participants) was chosen based on recommendation for sampling in pilot studies where little is known about treatment response rates [33]. Baseline demographic data including age, sex, race, level of intellectual quotient (IQ), ASD diagnostic sub-type, concomitant medications, and clinical ratings (CGI-S, ABC, SRS, and VABS-II scores) was compiled, and means and percentages were calculated to describe the NAC and placebo groups. Baseline demographic differences between groups were tested using chi-square tests (categorical data) and independent sample *t* test (continuous data) using a two-tailed *p* value of 0.05 for the alpha.

For the CGI-I primary outcome measure, differences between the two treatment groups were tested at weeks 4, 8, 12 using chi-square tests. For the CGI-S secondary outcome measure, a chi-square test was employed to examine change from baseline to week 12 by creating two CGI-S categories: (1) those subjects whose severity score decreased (i.e., clinically improved) by at least one point from baseline to week 12 and (2) those subjects whose scores remained the same during the same period. For the ABC, SRS, VABS-II, and oxidative stress biomarkers, differences between the groups for change in each outcome measure over the course of the study were tested using multi-level modeling (i.e., we examined if there were differences between the two groups in terms of change in the outcome scores over the course of the study). For each outcome, time was modeled at level-one as a random effect with treatment group entered as a level-two fixed effect. The cross-level interaction between group and time tested the differences between the two groups in the change over time using maximum likelihood estimation with robust standard errors with Mplus 5.21 [34] (baseline, week 4, week 8, and week 12 data was used for the ABC and SRS; baseline and week 12 data was used for the VABS-II and oxidative stress biomarkers). Since there is no method to directly calculate the effect size of the between-subject effects on the within-subject effects for multilevel modeling, we calculated an effect size of the difference between the groups on the mean differences between baseline and week 12 for the groups. Specifically, a Cohen’s *d* was calculated by subtracting the mean score at week 12 from the score at baseline for each group, and a difference score was calculated by subtracting the change scores between the groups. This difference score was then divided by the pooled standard deviation of the change scores. Independent sample *t* tests were used to compare the levels of oxidative markers between NAC and placebo groups at baseline and week 12. For all types of tests, a one-tailed *p* value of 0.05 was used as the alpha. A one-tailed test was used based on the a priori hypothesis that the NAC group would have greater change in clinical ratings and oxidative stress markers over the course of the study than the placebo group. We did not correct for multiple comparisons given the pilot nature of the work.

Adverse event data was compiled to describe the NAC and placebo groups. Separate analyses were conducted for the vital sign measurements, CMP, and CBC employing the same method as the treatment analyses, though only two time points, screen and week 12, were evaluated.

## Results

Thirty-one participants initially enrolled in the trial; however, six participants did not complete the study. Three participants were lost to follow-up after week 4 (two NAC and one placebo), and three withdrew due to irritability (NAC), diarrhea and encopresis (placebo), and defiant and self-injurious behavior (placebo), respectively. At baseline, data for all 31 enrolled subjects was employed in the analysis. Post-baseline, only data for the 25 completers was analyzed, as there is no method for imputing data for multi-level models.

At baseline, participants ranged in age from 4 to 12 years. The average age was 7.6 years (*SD* = 2.5, *n* = 16) for the NAC group and 8.2 years (*SD* = 2.9, *n* = 15) for the placebo group (Table 1). The majority of the participants were male (>75 %), white (>90 %), and had an IQ above 85. For the NAC group, the ASD diagnostic sub-types were primarily autistic disorder (43.8 %) and PDD-NOS (43.8 %) while the placebo group included primarily those with autistic disorder (46.7 %) and Asperger’s disorder (33.3 %). On the CGI-S scale, the majority of the participants in both groups (>85 %) were rated as either 5 “markedly” or 6 “severely ill”. There was no statistically significant baseline difference between groups for any clinical ratings (ABC, SRS, VABS-II), with the exception of a trend toward significantly higher score on the ABC stereotypy subscale in the placebo group (*t* value = 1.85, 0.07; NAC *M* = 4.75, *SD* = 3.3; placebo *M* = 7.00, *SD* = 4.9). Additionally, there was no significant difference in type or number of baseline concomitant medications between groups (all *p*s > 0.11).

**Table 1:** Demographic data and baseline characteristics

	Groups				Test of differences
	NAC		Placebo		
Characteristics	*n*/total	%	*n*/total	%	Chi-square^a^
Gender—male	12/16	75.0	12/15	80.0	0.11
Race—White	16/16	100.0	14/15	93.3	1.01
Diagnosis					
Autistic disorder	7/16	43.8	7/15	46.7	0.03
Asperger’s disorder	2/16	12.5	5/15	33.3	1.92
PDD-NOS	7/15	43.8	3/15	20.0	1.98
CGI-S					
Marked (5)	6/16	37.5	9/15	60.0	1.57
Severe (6)	9/16	56.3	6/15	40.0	0.82
Extreme (7)	1/16	6.3	0/15	0.0	0.97
Concomitant medication (y/n)	6/16	37.5	10/15	66.7	2.64
Medication types					
Psychostimulants	3/16	18.7	3/15	20.0	0.01
Alpha 2 agonists	1/16	6.2	2/15	13.3	0.44
Antipsychotics	4/15	25.0	5/15	33.3	0.26
Sleep aids	6/16	37.5	2/15	13.3	2.36
Antidepressants	1/16	6.2	3/15	20.0	1.30
Antiepileptic medication	2/16	12.5	0/15	0.0	2.01
	Mean	*SD*	Mean	*SD*	*t* test^b^
Age	7.63	2.5	8.20	2.9	0.59
Full scale IQ	86.27	21.8	87.43	11.7	0.18
Number of concomitant medications per participant	1.20	1.8	0.94	1.0	0.51

^a^Two-tailed significance ^+^ *p* < .10; *p < .05. No *p* values for these chi-squares reached significance at .05 and ranged from .11 to .87

^b^Two-tailed significance ^+^ *p* < .10; *p < .05 and *p*s ranged from .56 to .94

*NAC N*-acetylcysteine, *PDD-NOS* pervasive developmental disorder not otherwise specified, *CGI-S* clinical global impression severity scale

The average daily dose of NAC at week 12 was 56.2 mg/kg (*SD* = 9.7) with dose ranging from 33.6 to 64.3 mg/kg. Overall, NAC was well tolerated by study participants. The majority of adverse events occurred only once, and the frequency of adverse events was so low that comparisons between groups could not be conducted (Table 2). Overall, upper respiratory symptoms were the most commonly reported event for both groups (NAC *n* = 10, 62.5 %; placebo *n* = 6, 40.0 %). The next most frequent events were headaches (NAC *n* = 3, 18.7 %; placebo *n* = 3, 20.0 %), stomachache (NAC *n* = 2, 12.5 %; placebo *n* = 2, 13.3 %), and fever (NAC *n* = 3, 18.7 %; placebo *n* = 1, 6.7 %). The majority of reported adverse events were mild, with only five (6.7 %) reported as moderate. No severe adverse events were reported. There were no significant differences found between the groups for changes from screen to week 12 on vital signs or safety lab values (all *p* > 0.10).

**Table 2:** Frequency of adverse effects

	NAC (*n* = 16)			Placebo (*n* = 15)		
Adverse effects	Mild	Mod	Severe	Mild	Mod	Severe
URI	9	1	0	5	1	0
Headache	3	0	0	2	1	0
Stomachache	2	0	0	2	0	0
Fever	2	1	0	0	0	0
Irritability	2	0	0	1	0	0
Insomnia	2	0	0	0	0	0
Otitis media	1	1	0	1	0	0
Increased enuresis	1	0	0	1	0	0
Interrupted sleep	1	0	0	1	0	0
Localized rash	1	0	0	1	0	0
Nausea	1	0	0	1	0	0
Stereotypy	0	0	0	2	0	0

The table only lists adverse effects occurring ≥2 times across the samples

Placebo group reported one moderate case of the following adverse effects: defiant behavior, hives, itchy skin, and threats to hurt self

Placebo group reported one mild case of the following adverse effects: accidental injury (arm), constipation, diarrhea, dry mouth, emotional outburst, encopresis, increased hyperactivity, self-injurious behavior (skin picking), sinus infection, skin infection, sore throat, urinary tract infection, and weight gain

NAC group reported one mild case of the following adverse effects: aggression, appetite increase, behavior worse, change in speech, early morning awakening, edema, medical or surgical procedure, swollen neck/lymph nodes, and tic

*NAC N*-acetylcysteine, *Mod* moderate, *URI* upper respiratory infection

### Primary and secondary outcomes

There was no statistically significant difference between the NAC and the placebo groups at week 4 (*p* > 0.60), week 8 (*p* > 0.79), or week 12 (*p* > 0.69) on the CGI-I primary outcome measure (Table 3). At each time period, at least half of all participants (≥50 %) were rated as having no change. On the CGI-S secondary outcome measure, no participants were noted to have increased scores suggesting clinical worsening of symptoms. There were also no differences between the NAC and placebo groups for those whose severity scores decreased from baseline to week 12 (χ^2^ = 0.43, *p* = 0.40; NAC 46.2 %, *n* = 6; placebo 33.3 %, *n* = 4). On the ABC, SRS, and VABS-II secondary outcome measures, the employed models found no significant differences between groups in change from baseline to week 12 (all *p*s > 0.13 (Table 4)). Using the cutoff of a Cohen’s *d* of 0.25 for small, 0.50 for medium, and 0.80 for large, 100 % of the effects were ≤ the small effect size [35]. For all of the models that had a small effect size, the placebo group consistently had the larger average decrease in score.

**Table 3:** Clinical Global Impression-Improvement scale score, NAC vs. placebo

	Groups				Test of differences
	NAC		Placebo		
	*n*/total	%	*n*/total	%	Chi-square^a^
Week 4					0.28
Response	4/15	26.7	5/14	35.7	
No response	11/15	73.3	9/14	64.3	
Week 8					0.07
Response	5/13	38.5	4/12	33.3	
No response	8/13	61.5	8/12	66.7	
Week 12					0.15
Response	6/12	50.0	5/11	45.5	
No response	6/12	50.0	6/11	54.5	

^a^One-tailed significance ^+^ *p* < .10; *p < .05. No *p* values for these chi-squares reached significance at .05 and ranged from .60 to .79

Response = very much improved (1) or much improved (2); no response = minimally improved (3), no change (4), minimally worse (5); no participants scored much worse (6) or very much worse (7)

*NAC N*-acetylcysteine

**Table 4:** Means, standard deviations, and differences in change scores between NAC and Placebo groups

	NAC				Placebo						
	Baseline		12 Weeks		Baseline		12 Weeks			Group with largest change^b^	Cohen’s *d*
	M	SD	M	SD	M	SD	M	SD	*z* test^a^		
ABC											
Hyperactivity	20.6	12.4	17.4	16.4	22.6	10.5	15.1	10.8	1.04	PLB	0.34
Speech	3.9	2.8	4.1	3.9	5.7	2.9	5.14	3.5	0.66	PLB	0.24
Irritability	17.0	11.8	14.9	14.0	18.3	9.2	12.0	7.3	1.09	PLB	0.40
Lethargy	13.8	8.1	10.0	7.1	14.0	10.5	7.9	4.7	1.15	PLB	0.31
Stereotypy	4.8	3.3	3.9	5.1	7.0	4.9	5.8	4.0	0.56	PLB	0.09
SRS											
Total score	108.5	20.7	85.8	34.9	109.1	16.5	89.1	26.3	0.32	NAC	0.11
VABS-II											
Comm	85.9	32.1	88.5	32.5	89.4	32.2	95.0	30.3	0.85	PLB	0.09
Daily Liv. Sk.	93.1	33.7	99.0	37.1	87.6	28.1	98.6	33.4	0.17	PLB	0.16
Socialization	66.8	18.8	71.7	18.7	68.3	20.7	75.9	21.0	0.87	PLB	0.14
Composite	61.3	19.4	62.9	17.1	53.6	15.1	60.5	18.8	1.08	PLB	0.30
Levels in whole blood (uM)											
GSH	484.8	212.7	780.3	220.6	474.5	191.6	640.4	190.9	1.74*	NAC	0.64
GSSG	12.0	2.5	16.7	10.5	12.8	5.0	12.5	4.4	1.37^+^	NAC	0.88
GSH/GSSG	43.4	25.6	53.5	16.8	45.2	31.8	59.6	27.0	0.34	PLB	0.17
Homocysteine	4.3	6.1	9.9	13.7	1.9	1.2	5.6	6.8	0.41	NAC	0.27
DNA damage											
DNA strand breakage	0.7	0.1	0.7	0.3	0.6	0.2	0.6	0.1	0.38	EVEN	0.00
Oxidative DNA damage	0.9	0.2	1.2	0.4	1.1	0.5	1.2	0.6	0.95	NAC	0.45

^a^One-tailed significance ^+^ *p* < .10; *p < .05. *Z* test of the effect of between level variable of group on the within-level variable of time testing if the change from baseline to week 12 is different between groups. No one-tailed *p* values for these *z* tests reached significance at .05 and ranged from .13 to .50

^b^Identifies which group had the largest change from baseline to week 12

*NAC N*-acetylcysteine, *M* mean, *SD* standard deviation, *ABC* Aberrant Behavior Checklist, *PLB* placebo, *SRS* social responsiveness scale, *VABS-II* Vineland adaptive behavior scale 2nd edition, *Comm* communication, *Liv. Sk*. living skills, *GSH* glutathione, *GSSG* oxidative glutathione disulfide

### Oxidative stress biomarkers

Baseline (pre-treatment) levels of the oxidative markers including GSH, GSSG, GSH/GSSG ratio, DNA strand break and oxidative damage, and blood homocysteine were not significantly different between the NAC and placebo groups (*p* > 0.05 for all). At week 12, the GSH level in blood was significantly higher in the NAC group compared to placebo (780.3 vs. 640.4 μM; *p* < 0.05, Table 4). The GSSG level increased in the NAC treatment group, however with only marginal significance in comparison with the placebo group (16.7 vs. 12.5 μM; *p* = 0.09, Table 4). Using the Cohen’s *d* cutoff employed above for effect sizes, the size of the differences for the significant and the marginally significant effects were large- and medium-sized effects, respectively. For the GSH/GSSG ratio, strand break and oxidative damage of DNA, as well as blood homocysteine, there were no significant differences between the NAC and placebo groups from baseline to week 12 (*p*s > 0.16).

## Discussion

This study was designed to evaluate the safety and efficacy of NAC targeting core social impairment in youth with ASD. The results of this randomized, placebo-controlled trial indicate that NAC treatment was well tolerated by study participants, had the expected effect of boosting GSH production in peripheral blood, but had no significant impact on the core social impairment of ASD when compared to placebo treatment. The results of this study do not support the use of NAC for treatment of core social impairment of ASD, though the health impact of the resultant increase on GSH remains unclear. In addition, the study did not note other behavioral improvements with NAC use such as the reduction in irritability reported by Harden et al. (2012) [13].

Interpretation of these results must be taken in context of the study’s limitations. Participant diagnoses were made by study physicians with expertise in ASD corroborated by the ADI-R similar to methods employed in other ASD drug studies [36]. Nevertheless, administration of a research reliable Autism Diagnostic Observation Schedule would have added to the validity of diagnoses in this study. Furthermore, limitation of the study to just participants with diagnosis of autistic disorder would have lessened the heterogeneity of our sample. Our study population was potentially biased toward individuals with higher level of functioning, as the majority of participants had IQ above 85. This limits the generalizability of these study results to the broader population of individuals with ASD. This higher functioning study population may have also contributed to lack of improvement in irritability noted in this study. The baseline ABC-I of study participants was 17.0 (*SD* 11.8) in the NAC group and 18.3 (*SD* 9.2) in the placebo group, which is slightly below the standard cutoff ABC-I score of 18 used in drug studies targeting ASD-associated irritability [36]. However, the baseline ABC-I scores in our study are higher than those in the Hardan study which demonstrated improvement irritability with NAC treatment [13], so the impact this had on our study outcome is unclear. Additionally, the small sample size combined with the inherent significant placebo response rates in ASD core symptom trials enhances type II error potential thus rendering the project potentially underpowered to detect meaningful change. The small sample size also reduces the ability to effectively correlate treatment response with baseline oxidative stress markers or with change in such markers with treatment.

The NAC formulation employed in this study was a powder which was encapsulated by the investigative pharmacy and stored under normal pharmacy conditions. No studies of impurity were completed on this compound, and no analyses were completed to assess its stability. Furthermore, this packaging was different than the method employed by Hardan et al. (2012) who utilized an individual foil packaging method to protect the integrity of the active ingredient against oxidation [13]. While this difference could have in theory reduced the potency of our formulation, the biologic impact of our treatment was clear given the significant NAC-associated increase in GSH levels at week 12. It is unclear; however, if the change in oxidative markers and potential clinical impact may have differed had we employed an individual foil packaging method. Further, whether an increase in peripheral blood GSH levels, and the NAC formulation employed in our study, translates to clinically significant CNS antioxidant activity is unclear as there remains debate in the field regarding the ability of NAC to efficiently cross the BBB [10]. In future studies, alterations to NAC formulation including esterification of the carboxyl group producing *N*-acetylcysteine ethyl ester or creation of the amide derivative *N*-acetylcysteine amide may increase this medication’s oral bioavailability, BBB permeability, and therefore therapeutic potential [10].

## Conclusions

The results of this study suggest that NAC treatment does not have significant impact on the core social impairment of ASD. However, the small study size limits interpretation of these results. Additional larger-scale study powered to predict treatment response based on baseline peripheral oxidative markers may be indicated. Future studies may also wish to consider the use of a NAC formulation with improved bioavailability and BBB permeability.

## Abbreviations

ABC: Aberrant Behavior Checklist
ADI-R: Autism Diagnostic Inventory Revised
ASD: autism spectrum disorder
BBB: blood-brain barrier
CBC: complete blood cell count
CGI-I: Clinical Global Impression Improvement
CGI-S: Clinical Global Impression Severity
CMP: comprehensive metabolic panel
GSH: glutathione
GSSG: glutathione disulfide
HPLC: high-performance liquid chromatography
IQ: intellectual quotient
IUSM: Indiana University School of Medicine
M: mean
N: number
NAC: *N*-acetylcysteine
PDD NOS: pervasive developmental disorder not otherwise specified
SD: standard deviations
SRS: Social Responsiveness Scale
VABS-II: Vineland Adaptive Behavior Scale 2nd Edition

## References

[1] Kanner L (1943). Autistic disturbances of affective contact. Nerv Child.

[2] Reichow B, Steiner AM, Volkmar F (2013). Cochrane review: social skills groups for people aged 6 to 21 with autism spectrum disorders (ASD). Evidence-based child health : a Cochrane review journal.

[3] Autism Spectrum Disorder, Data and Statistics [http://www.cdc.gov/ncbddd/autism/data.html]. Accessed 4/3/2016.

[4] James SJ, Melnyk S, Jernigan S, Cleves MA, Halsted CH, Wong DH, Cutler P, Bock K, Boris M, Bradstreet JJ (2006). Metabolic endophenotype and related genotypes are associated with oxidative stress in children with autism. Am J Med Genet B Neuropsychiatr Genet.

[5] Erickson CA, Posey DJ, Stigler KA, McDougle CJ, Heresco-Levy U (2008). Glutamatergic function in autism. Glutamate in Neuropsychiatric Disorders.

[6] Kern JK, Jones AM (2006). Evidence of toxicity, oxidative stress, and neuronal insult in autism. J Toxicol Environ Health B Crit Rev.

[7] James SJ, Rose S, Melnyk S, Jernigan S, Blossom S, Pavliv O, Gaylor DW (2009). Cellular and mitochondrial glutathione redox imbalance in lymphoblastoid cells derived from children with autism. FASEB J.

[8] Deepmala, Slattery J, Kumar N, Delhey L, Berk M, Dean O, Spielholz C, Frye R (2015). Clinical trials of N-acetylcysteine in psychiatry and neurology: a systematic review. Neurosci Biobehav Rev.

[9] Olsson B, Johansson M, Gabrielsson J, Bolme P (1988). Pharmacokinetics and bioavailability of reduced and oxidized N-acetylcysteine. Eur J Clin Pharmacol.

[10] Bavarsad Shahripour R, Harrigan MR, Alexandrov AV (2014). N-acetylcysteine (NAC) in neurological disorders: mechanisms of action and therapeutic opportunities. Brain Behav.

[11] Berk M, Malhi GS, Gray LJ, Dean OM (2013). The promise of N-acetylcysteine in neuropsychiatry. Trends Pharmacol Sci.

[12] Aman MG, Singh NN, Stewart AW, Field CJ (1985). The Aberrant Behavior Checklist: a behavior rating scale for the assessment of treatment effects. Am J Ment Defic.

[13] Hardan AY, Fung LK, Libove RA, Obukhanych TV, Nair S, Herzenberg LA, Frazier TW, Tirouvanziam R (2012). A randomized controlled pilot trial of oral N-acetylcysteine in children with autism. Biol Psychiatry.

[14] Ghanizadeh A, Moghimi-Sarani E (2013). A randomized double blind placebo controlled clinical trial of N-Acetylcysteine added to risperidone for treating autistic disorders. BMC Psychiatry.

[15] Nikoo M, Radnia H, Farokhnia M, Mohammadi MR, Akhondzadeh S (2015). N-acetylcysteine as an adjunctive therapy to risperidone for treatment of irritability in autism: a randomized, double-blind, placebo-controlled clinical trial of efficacy and safety. Clin Neuropharmacol.

[16] Ghanizadeh A, Derakhshan N (2012). N-acetylcysteine for treatment of autism, a case report. J Res Med Sci.

[17] Marler S, Sanders KB, Veenstra-VanderWeele J (2014). N-acetylcysteine as treatment for self-injurious behavior in a child with autism. J Child Adolesc Psychopharmacol.

[18] Melnyk S, Fuchs GJ, Schulz E, Lopez M, Kahler SG, Fussell JJ, Bellando J, Pavliv O, Rose S, Seidel L (2012). Metabolic imbalance associated with methylation dysregulation and oxidative damage in children with autism. J Autism Dev Disord.

[19] Guy W (1976). ECDEU assessment manual for psychopharmacology, Publication No. 76-338.

[20] American, Psychiatric Association (1994). Diagnostic and Statistical Manual of Mental Disorders.

[21] Lord C, Rutter M, Le Couteur A (1994). Autism Diagnostic Interview-Revised: a revised version of a diagnostic interview for caregivers of individuals with possible pervasive developmental disorders. J Autism Dev Disord.

[22] Roid G, Miller L (1997). Leiter International Performance Scale—revised.

[23] Constantino J, Gruber C (2005). Social Responsiveness Scale.

[24] Sparrow S, Cicchetti D, Balla DA (2008). Vineland Adaptive Behaviors Scales.

[25] Research Units on Pediatric Psychopharmacology Autism N (2002). Risperidone in children with autism and serious behavioral problems. N Engl J Med.

[26] McDougle CJ, Scahill L, Aman MG, McCracken JT, Tierney E, Davies M, Arnold LE, Posey DJ, Martin A, Ghuman JK (2005). Risperidone for the core symptom domains of autism: results from the study by the autism network of the research units on pediatric psychopharmacology. Am J Psychiatry.

[27] Buxbaum JD, Bolshakova N, Brownfeld JM, Anney RJ, Bender P, Bernier R, Cook EH, Coon H, Cuccaro M, Freitag CM (2014). The Autism Simplex Collection: an international, expertly phenotyped autism sample for genetic and phenotypic analyses. Molecular autism.

[28] Melnyk S, Pogribna M, Pogribny I, Hine RJ, James SJ (1999). A new HPLC method for the simultaneous determination of oxidized and reduced plasma aminothiols using coulometric electrochemical detection. J Nutr Biochem.

[29] Vester B, Rasmussen K (1991). High performance liquid chromatography method for rapid and accurate determination of homocysteine in plasma and serum. Eur J Clin Chem Clin Biochem.

[30] Probst R, Brandl R, Blumke M, Neumeier D (1998). Stabilization of homocysteine concentration in whole blood. Clin Chem.

[31] Collins AR, Dobson VL, Dusinska M, Kennedy G, Stetina R (1997). The comet assay: what can it really tell us?. Mutat Res.

[32] Singh NP, McCoy MT, Tice RR, Schneider EL (1988). A simple technique for quantitation of low levels of DNA damage in individual cells. Exp Cell Res.

[33] Julious S (2005). Sample size of 12 per group rue of thumb for a pilot study. Pharm Stat.

[34] Muthen LK, Muthen BO (1998). Mplus User's Guide 6th Edition.

[35] Cohen J (1988). Statistical power analysis for the behavioral sciences.

[36] McCracken JT, McGough J, Shah B, Cronin P, Hong D, Aman MG, Arnold LE, Lindsay R, Nash P, Hollway J (2002). Risperidone in children with autism and serious behavioral problems. N Engl J Med.

